# Secondary Attack Rate of Tuberculosis in Urban Households in Kampala, Uganda

**DOI:** 10.1371/journal.pone.0016137

**Published:** 2011-02-14

**Authors:** Christopher C. Whalen, Sarah Zalwango, Allan Chiunda, LaShaunda Malone, Kathleen Eisenach, Moses Joloba, W. Henry Boom, Roy Mugerwa

**Affiliations:** 1 Department of Epidemiology and Biostatistics, College of Public Health, University of Georgia, Athens, Georgia, United States of America; 2 Tuberculosis Research Unit, Makerere University, Kampala, Uganda; 3 Department of Medicine, Case Western Reserve University, School of Medicine and University Hospitals of Cleveland, Cleveland, Ohio, United States of America; 4 Department of Pathology, University of Arkansas for Medical Sciences, Little Rock, Arkansas, United States of America; 5 Department of Medicine, Makerere University Medical School, Kampala, Uganda; University of Stellenbosch, South Africa

## Abstract

**Background:**

Tuberculosis is an ancient disease that continues to threaten individual and public health today, especially in sub-Saharan Africa. Current surveillance systems describe general risk of tuberculosis in a population but do not characterize the risk to an individual following exposure to an infectious case.

**Methods:**

In a study of household contacts of infectious tuberculosis cases (n = 1918) and a community survey of tuberculosis infection (N = 1179) in Kampala, Uganda, we estimated the secondary attack rate for tuberculosis disease and tuberculosis infection. The ratio of these rates is the likelihood of progressive primary disease after recent household infection.

**Results:**

The secondary attack rate for tuberculosis disease was 3.0% (95% confidence interval: 2.2, 3.8). The overall secondary attack rate for tuberculosis infection was 47.4 (95% confidence interval: 44.3, 50.6) and did not vary widely with age, HIV status or BCG vaccination. The risk for progressive primary disease was highest among the young or HIV infected and was reduced by BCG vaccination.

**Conclusions:**

Early case detection and treatment may limit household transmission of *M. tuberculosis*. Household members at high risk for disease should be protected through vaccination or treatment of latent tuberculosis infection.

## Introduction

Tuberculosis is a disease that is both curable and preventable, yet still poses a threat to personal and public health today, especially in developing countries. In most countries, the burden of tuberculosis is monitored by rates of disease obtained through surveillance systems that rely on passive case finding and centralized reporting. This type of surveillance is subject to the ecologic fallacy because it describes the average risk of tuberculosis in a population but does not characterize the risk to an individual following exposure to an infectious case. For an individual living in an area endemic for tuberculosis, the latter risk may be of greater relevance.

In a setting endemic for tuberculosis, such as Sub-Saharan Africa, one cannot always determine whether heightened risk for tuberculosis results from increased frequency of exposure to infectious cases due to the high prevalence of disease, enhanced risk of acquiring infection once exposed, or increased risk of disease once infected. The secondary attack rate (SAR), which measures the probability of disease transmission to an individual in the context of a defined exposure [1], [2], may be used to tease apart these component risks among household contacts. Although the SAR is most often applied to infectious diseases with short incubation periods in well-defined social networks, such as households, schools, and hospitals [1], [3]–[5], its methods may be extended to include chronic infectious diseases, such as tuberculosis, with the use of modern molecular techniques to identify and track strains.

In this report, we adapt classic concepts of SAR to tuberculosis and derive new ways to determine the SAR for both tuberculosis infection and disease, and to estimate the risk of developing tuberculosis after household exposure.

## Methods

This study was approved by the Ugandan Council for Science and Technology and the Institutional Review Board at the University Hospitals of Cleveland. Informed consent was obtained from adults, assent from adolescents with permission from parents or guardians, and consent from parents or guardians for children. All consent was obtained in writing.

To study the dynamics of *M. tuberculosis* transmission and active tuberculosis in African households, we performed a longitudinal study of tuberculosis (sputum smear-positive for acid fast bacilli) in 497 index cases and their household contacts (n = 1918, Figure 1). Tuberculosis cases were identified at the Tuberculosis Treatment Center of Mulago Hospital in Kampala, Uganda [6]. Household contacts were identified through household contact tracing performed within 4 weeks of the initial diagnosis of tuberculosis in the index case. Contacts were followed for two years from the time of diagnosis in the index case and were evaluated at 6 month intervals for tuberculosis disease. These evaluations included history and physical examination; contacts identified as tuberculosis suspects were further evaluated with sputum microscopy and culture, chest radiography, and HIV serostatus. A similar approach was used for sick visit evaluations. Tuberculin skin testing was repeated three months after household evaluation to include recent skin test converters. Of 442 contacts with a tuberculin skin test (TST)<5 mm at baseline, 380 contacts (86%) were available for repeat evaluation.

**Figure 1:**
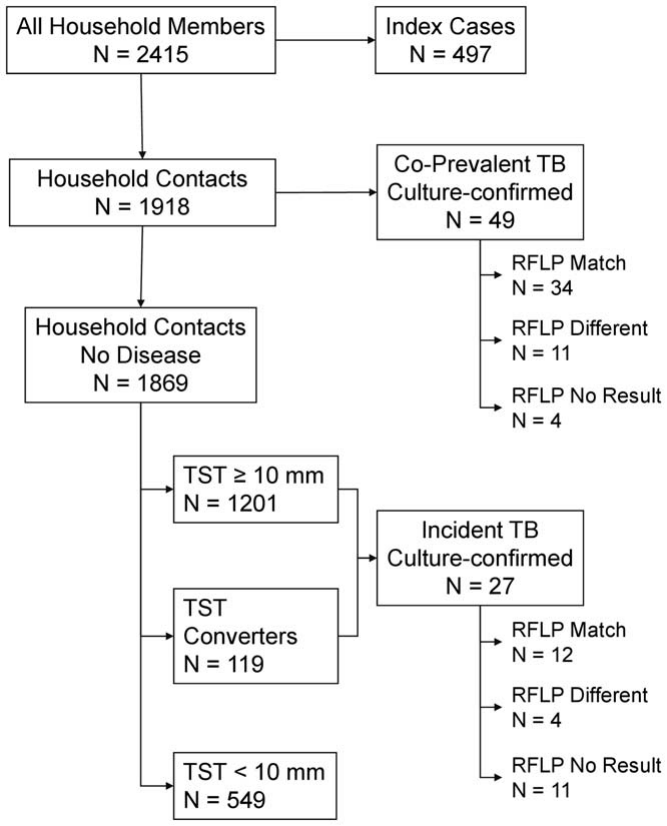
Distribution of tuberculosis and latent tuberculosis infection among 2415 households in Kampala Uganda, 1995–2004.

To measure the prevalence of tuberculosis infection in households without active cases, we performed a cross-sectional study of 200 neighborhood control households without cases of active tuberculosis and enrolled 1179 people residing in the same or adjacent neighborhoods. Neighborhood control households were identified by selecting a neighboring village to the index household within the same or adjacent parish, and then by randomly selecting households for the study either from a pre-assembled list of households in the village, if available, or by recruiting consecutive households along a road or path. Households were eligible to be controls if no case of tuberculosis was present in the household for at least one year, at least one member in the household was within 5 years of age as the index case, and the household contained two or more members. By choosing adjacent or neighboring parishes to the index households, community controls were matched to the index households for socioeconomic status and underlying level of community transmission.

In each index case and neighborhood household, we evaluated all members for latent tuberculosis infection and active tuberculosis using standard clinical methods [7] within four weeks of household evaluation and estimated the age-specific prevalence of latent tuberculosis infection and active disease. Co-prevalent tuberculosis was defined as a tuberculosis case occurring within three months of the initial diagnosis in the index case; incident tuberculosis was defined as a case of disease occurring after three months [6]. Latent tuberculosis infection was measured using purified protein derivative (Tubersol) and the Mantoux method. A criterion for a positive test of 10 mm was used to minimize misclassification from previous BCG vaccination [8]. Contacts who converted the TST to positive with 3 months were considered to be infected at baseline [9]. The presence of a BCG scar was assessed by a trained health care provider and verified with medical records where possible. Tuberculosis suspects were evaluated with medical history, physical examination, sputum microscopy and culture, and chest x-ray [6].

To characterize the strains of *M. tuberculosis* in households, sputum samples were obtained from the 76 household contacts with culture-confirmed tuberculosis and their index cases. Isolates of *M. tuberculosis* from 61 pairs (80%) were analyzed using restriction fragment length polymorphisms [10] (RFLP) to determine strain type. In 15 pairs, an isolate from either the index case or contact was not available because of contamination or failure to grow. Isolates of *M. tuberculosis* were considered to be matched if they had: (1) more than five copies of IS6110 and the fragments showed 100 percent match at a band deviation of 2.5 percent or less; (2) less than six copies of IS6110 and the fragments were 100 percent matched and the isolates showed identical PGRS patterns [11]. A secondary case of tuberculosis was defined as a contact case who had disease with the same strain of *M. tuberculosis* as the index case as determined by the RFLP pattern of both isolates. For the purposes of this analysis, we assume that infection in the index and contact cases did not occur through a common source case outside of the household.

To apply the concepts of the SAR to tuberculosis, we decomposed the attack rate into two parts that reflect the natural history of the disease [12] and then derived methods to estimate the SAR for tuberculosis disease and infection separately. In the natural history of tuberculosis, infection with *M. tuberculosis* must first occur in a susceptible individual after one or more exposures to an infectious index case. Once infection is established, active disease may ensue depending on host immune response and virulence properties of the pathogen. The SAR for tuberculosis disease (SAR_D_) may be thought of as the product of the SAR for infection with *M. tuberculosis* from the index case (SAR_I_) and the probability of developing disease within a specified time interval following infection (*p*_D|I_):
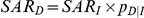
The SAR for tuberculosis disease was estimated directly through contact investigations by determining the proportion of household contacts that had or developed tuberculosis within 24 months of the diagnosis in the index case and shared the same strain of *M. tuberculosis* as the index case using RFLP analysis. For comparison, the SAR for disease was calculated separately using all contact cases regardless of strain type. Since we were not able to obtain RFLP results on 15 culture-confirmed contact cases, we estimated the total number of matched strains as the sum of observed and expected matches. Expected matches were estimated for index-case isolate pairs without RFLP results according to the proportions observed in pairs with RFLP patterns.

The SAR for tuberculosis infection in household contacts is the probability of infection by the same strain of *M. tuberculosis* as the infectious index case during the exposure period. Since it is not possible to know the strain producing a latent tuberculosis infection, we estimated the SAR for infection as the difference in age-specific prevalence of latent infection between the household contacts and community controls (Appendix S1). The prevalence difference estimates the additional risk for latent infection associated with living in a house of an infectious index case. With the SAR for tuberculosis disease and infection estimated, the probability of progressive primary tuberculosis given recent household infection (*p*_D_) is the ratio of the SAR for disease to the SAR for infection.

## Results

Household contacts (n = 1918) and community members (n = 1179) were similar as regards age, gender, vaccination with BCG, level of crowding in the household, type and location of residence. Among the 1918 household contacts, 114 cases of tuberculosis were identified, of which 76 cases (67%) were confirmed by culture. Culture-confirmed disease was present in 28 of 55 (53%) children younger than 5 years, 7 of 10 (70%) children 5 to 15 years, and 40 of 49 (82%) contacts older than 15 years. Of the 76 culture-confirmed cases, 49 cases were co-prevalent cases, the remaining 27 were incident cases occurring during the 24 month follow-up period. RFLP analysis was performed on 61 of the 76 isolates (80%). Overall, the RFLP pattern of contact cases matched the pattern of index cases in 46 of 61 pairs (75%; Table 1). In the remaining 15 pairs of index and contact cases, the RFLP pattern did not match; these isolate pairs are distributed among children, HIV seropositive, and BCG vaccinated contacts (Table 1). HIV serostatus was not known for 262 contacts; 2 cases of tuberculosis with a matched isolate occurred among these contacts. BCG vaccination status was not known or was uncertain for 70 contacts; 1 case of tuberculosis with a matched isolate occurred among these contacts.

**Table 1:** Estimates of the secondary attack rate of tuberculosis in 1918 household contacts in Kampala, Uganda, 1995–2004.

Characteristic	Category	No. at Risk	No. Positive Culture Cases	No. without RFLP	No. RFLP Matched Isolates	Estimated No. Matched Isolates†	SAR - Tuberculosis (%)	95% CI
Overall		1918	76	15	46	57.3	3.0	2.2, 3.8
Age (y)	≤5	508	28	3	23	25.8	5.1	3.2, 7.0
	6–15	691	7	3	3	5.3	0.8	0.1, 1.4
	16–25	364	16	3	8	9.8	2.7	1.0, 4.4
	26–45	283	22	5	11	14.2	5.0	2.5, 7.6
	≥46	72	3	1	1	1.5	2.1	0, 5.4
	>5	1410	48	12	23	30.7	2.2	1.4, 2.9
HIV Status	HIV+	201	30	8	13	17.7	8.8	4.9, 12.7
	HIV−	1455	44	7	31	36.9	2.5	1.7, 3.3
BCG Vaccine	Yes	1349	46	6	32	36.8	2.7	1.9, 3.6
	No	499	27	7	13	17.6	3.5	1.9, 5.1

**Co-prevalent cases with the same finger print pattern as the index case. Since 15 cases did not have RLFP results, this number is estimated using the observed proportion (see methods) of RLFP matches. 46/61 observed matches; thus, 46/61*76 culture confirmed cases = 57.3 = 57.

† The total number of cases with matched RFLP patterns is the number of isolates with observed matches plus expected number of matches from isolates grown in culture but not analyzed with RFLP. Expected number of matches was estimated as the product of the observed proportion of matches and the number of pairs without RFLP results plus observed matches.

*HIV serostatus was not available in 262 (13.7%) of contacts. HIV serostatus was not measured in community control households; the general secondary attack rate for infection was therefore used to estimate risk of disease after household infection.

‡ Vaccination status missing or uncertain in 70 household contacts and 4 community members.

The overall SAR for disease using case pairs with matched RFLP patterns was 3.0% (95% confidence interval: 2.2, 3.8; Table 1). Without accounting for the strain types, the SAR for disease was 3.9%, an overestimation of 25%. The SAR for disease was bimodal according to age with the highest risk among children 5 years old or younger (5.1%) and among contacts 26 to 45 years old (5.0%), and the lowest risk among contacts 6 to 15 years old (0.8%; Table 1). The high level of SAR for disease in the age category 26–45 was attributable to HIV infection; when analyzing only the HIV seronegative contacts by age, the SAR for disease dropped in the age category to 2.7 (95% CI: 0.3, 5.0), whereas the rate of disease remained similar in the other age groups. In HIV-infected contacts the SAR for disease was 8.8%, whereas in HIV seronegative contacts, the rate was 2.5%. For contacts with BCG vaccination, the SAR for disease was 2.7% for contacts compared with 3.5% for contacts without vaccination.

**Figure 2:**
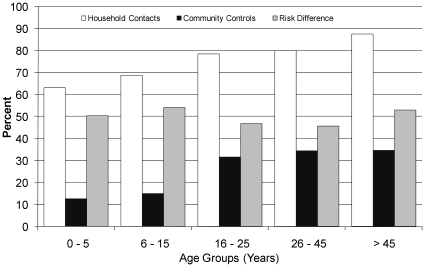
Prevalence of latent tuberculosis infection (TST≥10 mm) and risk difference according to age among household contacts and community controls in Kampala, Uganda, 1995–2004.

Of the 1918 contacts, 1201 contacts (63%) without disease had TST≥10 mm, 119 contacts (6%) converted to a positive TST within three months of initial evaluation, and 49 had co-prevalent disease (2.6%), yielding a total of 1369 contacts (71%) with infection at the time of household investigation. The prevalence of infection was greater for household contacts compared to community controls for all age categories (Table 2). The overall difference in prevalence of infection was 47.4% (95% confidence interval: 44.3, 50.6). Among the household contacts, the prevalence of tuberculosis infection increased with age from 63% in children 5 years and younger to 87.5% among older adults (Table 2, Figure 2). Among community members, the prevalence of tuberculosis infection increased with age from 12.6% in children 5 years and younger to 34.6% among older adults (Table 3, Figure 2). The age-specific prevalence difference ranged from 45.5 to 53.9% across the age groups but did not differ among age groups (test for linear trend, P = 0.91).

**Table 2:** Prevalence of tuberculosis infection and risk difference in tuberculosis infection between 1918 household contacts and 1179 community members according to age and BCG vaccination status in Kampala, Uganda, 1995–2004.

Characteristic	Category	Exposure	N	Number Infected	Infected (%)	Risk Difference	95% CI
Total		Contacts	1918	1369	71.4	47.4	44.3, 50.6
		Controls	1179	282	23.9		
Age	0–5	Contacts	508	320	63.0	50.3	44.5, 56.2
		Controls	253	32	12.6		
	6–15	Contacts	691	475	68.7	53.9	48.7, 59.2
		Controls	311	46	14.8		
	16–25	Contacts	364	285	78.3	46.7	39.8, 53.6
		Controls	275	87	31.6		
	26–45	Contacts	283	226	79.9	45.5	38.1, 52.9
		Controls	262	90	34.4		
	≥46	Contacts	72	63	87.5	52.9	39.9, 65.8
		Controls	78	27	34.6		
BCG Vaccine	Yes	Contacts	1349	935	69.3	47.4	43.6, 51.2
		Controls	793	174	21.9		
	No	Contacts	499	388	77.7	49.7	44.0, 55.5
		Controls	382	107	28.0		

‡ Vaccination status missing or uncertain in 70 household contacts and 4 community members.

*Defined as the sum of contacts with TS>10 mm within 3 months of household evaluation who do not have evidence of active tuberculosis.

**Table 3:** Estimate for progressive primary tuberculosis using the secondary attack rate (SAR) for tuberculosis disease and tuberculosis infection among 1918 household contacts of infectious index cases in Kampala, Uganda.

Characteristic	Category	SAR Tuberculosis Disease (%)	SAR Tuberculosis Infection (%)	Progressive Primary Tuberculosis (%)	95% CI
Overall		3.0	47.4	6.3	0, 13.3
Age	≤5	5.1	50.3	10.1	1.8, 18.4
	6–15	0.8	53.9	1.4	0, 4.6
	16–25	2.7	46.7	5.8	0, 12.5
	26–45	5.0	45.5	11.1	1.9, 20.2
	≥46	2.1	52.9	3.9	0, 9.2
	>5	2.2	47.4	4.6	0, 10.5
HIV Status	HIV+	8.8	47.4	18.6	7.51, 29.7
	HIV−	2.5		5.3	0, 11.8
BCG Vaccine	Yes	2.7	47.4	5.7	0, 12.5
	No	3.5	49.7	7.1	0, 14.2

Because BCG vaccination may confound the relation between household exposure to and infection with *M. tuberculosis*, we performed a stratified analysis based on BCG vaccination (Table 2). The prevalence of tuberculosis infection was greater among non-vaccinated contacts and controls compared with their vaccinated counterparts. Prevalence of infection was also greater in contacts than controls regardless of vaccination status. The prevalence difference in infection was similar regardless of BCG vaccination status.

The overall risk of progressive primary disease, that is the probability of developing disease after acquiring new infection with *M. tuberculosis* through household contact, was 6.3% (Table 3). Part of this elevated risk was carried by children 5 years old or younger who had a conditional risk of disease of 10.1% as compared with the risk of 4.6% in contacts older than 5 years. HIV infection in the household contact conferred highest absolute risk for progressive primary disease of 18.6%. The probability of disease was 20% lower in the vaccinated compared with the unvaccinated contacts.

## Discussion

In this study from an urban setting in East Africa, we found that, overall, the SAR for disease was 3% but that it varied according to age and HIV serostatus, as expected. The SAR for infection with *M. tuberculosis* was high, 47%, but it was similar across age groups, HIV status, and BCG vaccination, indicating parity in the risk for tuberculosis infection among household contacts. Thus, the observed variation in the SAR for disease was attributable not to the likelihood of acquiring new infection in the household but to the differing risks for progressive primary disease among newly infected household contacts.

The SAR of an infectious disease quantifies the risk of disease transmission to an individual in the context of a defined exposure [1], [13]. Formally, the SAR is the conditional probability of transmission of infection, or disease, to a susceptible. This analysis extends the classic model of the SAR for infectious diseases [1], [14] to tuberculosis in a household contact setting. By representing the natural history of tuberculosis as a two-stage process of infection followed by disease [12], and by evaluating household contacts where the exposure to an infectious case is known by design, we separate the risk for infection from the risk for disease, and thereby obtain separate estimates for the SAR for infection and the SAR for disease. Moreover, the ratio of these attack rates provides the likelihood of progressive primary disease resulting from recent household infection and adjusts for previous tuberculosis infection in contacts.

In the household contact setting, the SAR is used as a measure of risk for disease in the household and is estimated as the proportion of household members exposed who also develop disease within a specified time period. The validity of the SAR, however, depends on the degree of concordance of strain types between index and contact cases. Because some disease in households results from transmission outside the household contact network, failure to account for these cases overestimates the SAR for disease. Recent population-based studies from industrialized countries have shown that the strain of *M. tuberculosis* may differ between the index and contact cases in up to 30% of pairs. In this study, we observed a similar proportion of discordant pairs. In fact, in this setting, the SAR for disease would have been overestimated by 25% without verifying the strain-specific chain of transmission by RFLP analysis.

Tuberculosis has a long and variable latent period, sometimes lasting decades. To convey meaning about risk for disease, the SAR for disease must specify a time frame for the development of disease. In this study, the SAR for disease captured risk for two years after the diagnosis of the index case. By design, then, we estimated the risk for progressive primary disease after household exposure to an index case. The SAR captures the risk of disease after *exposure* to an infectious case but does not accurately estimate the risk of disease after acquiring *new infection*. As seen in this study, and in other household contact studies [15]–[18], not all exposed household contacts become infected. Since we estimated the SAR for infection to be 47%, the actual risk of developing disease after acquiring new infection is about twice the SAR for disease [18].

In this analysis, we merged the concepts of the SAR with those of disease prevalence [19] and multi-causal models [20]–[22] to estimate the SAR for tuberculosis infection in households. This method estimates SAR for infection by calculating the age-specific difference in prevalence of latent tuberculosis infection between household contacts and community members. This prevalence difference best approximates the SAR for infection when the annual risk of infection in the community is low or when the infectious period for the index case is short (Appendix S1). In this study, the median duration of cough, a surrogate for infectiousness, was 90 days [6], so with an annual risk of infection is as high as 3% per year [23], the prevalence difference overestimates the SAR by less than 1%. If we restrict our interest to a specific strain of *M. tuberculosis*, that is, the strain producing disease in the index case, then the prevalence difference is likely to be an excellent estimate of the SAR because in endemic settings, there are typically hundreds of circulating strains during any period of time [24]–[26] so the annual risk of infection from a specific strain in the community will be small.

This estimate of the SAR for infection carries other limitations and assumptions. Although the TST is the standard method for assessing infection with *M. tuberculosis*, it lacks sensitivity in the setting of immunosuppression (e.g., HIV infection or malnutrition) and specificity where BCG vaccination is widely used [27], [28]. Although HIV infection is endemic in Uganda and may cause false-negative TST results that may lead us to underestimate the SAR for infection, the HIV seroprevalence of 12% among contacts did not affect the prevalence difference (data not shown). To minimize false-positive misclassification of the TST results due to BCG vaccination, we used 10 mm as our criterion for a positive TST [8]. Some of the limitations of the TST may be mitigated by the use of interferon-γ release assays which may improve upon the specificity of the TST in the diagnosis of latent tuberculosis infection. The methods presented here can be readily modified to use the new immune-based assays in estimating secondary attack rates. In this analysis, we also estimated the average risk of infection as the difference in average age-specific prevalence of latent infection (i.e., the prevalence in household contacts compared with community members). At the individual level, these estimates may not apply because a given contact may have been previously infected and experience risks that differ from the overall average of that age group.

In the household of an infectious index case, the interactions between the contacts and index case are complex. The duration and intensity of exposure to the index case may depend on the familial relationship, traditional roles of caring for ill relatives, ability of the index case to cough, ventilation in the house, to name a few. Each discrete exposure is associated with a real but unknown probability of becoming infected. Since it is not feasible to measure the risk of infection for any single exposure to the index case, we used age-specific prevalence as a measure of the cumulative risk over time of the discrete and multiple exposures. We assume a binomial model, discrete exposures occurring randomly in time, and homogeneous mixing of household members.

In conclusion, we have combined modern molecular techniques with traditional epidemiologic methods to introduce a new approach for estimating the risk of tuberculosis following recent infection with *M. tuberculosis* in African households. This method shows that contact cases of tuberculosis often, but not always, shared the same strain of *M. tuberculosis* as the index case, despite high level of tuberculosis transmission in the community. The risk for tuberculosis infection resulting from household transmission in an urban African home is high. Since the risk of infection did not vary widely with age or previous BCG vaccination, the observed variability in progressive primary disease depended on characteristics such as age and immune status of the household contact. These observations highlight the importance of careful exposure history, especially in the context of drug-resistant tuberculosis, and early case detection and treatment to limit household transmission of *M. tuberculosis*. Furthermore, household members at high risk for disease must be protected through treatment of latent tuberculosis infection.

## Supporting Information

Appendix S1 (DOC)
