# Supplementary material for: Secondary Attack Rate of Tuberculosis in Urban Households in Kampala, Uganda
Source: PLoS One. 2011 Feb 14;6(2):e16137. doi: 10.1371/journal.pone.0016137 (PMC3038854; doi:10.1371/journal.pone.0016137)
Supplement: Appendix S1 — (DOC) [file pone.0016137.s001.doc]

Appendix

We propose an indirect method for estimating the SAR for tuberculosis infection by calculating the difference in age-specific prevalence [19,29] of tuberculosis infection, as measured by the tuberculin skin test, between household contacts and controls in the community.

For a person living in the community, the cumulative probability of infection with *M. tuberculosis* from birth to age *a* can be thought of as the probability of infection from birth to some intermediate age, *t*, plus the additional risk from age t to age a (Figure 1):


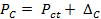


where *P_C_* is the cumulative probability of infection with *M. tuberculosis* from community transmission from birth to age a, *P_ct_* is the probability of infection from community transmission from birth to age t, and *∆_C_* is the probability of infection from age t to age *a* from community transmission.

For persons in the community who are exposed to an infectious tuberculosis case at age *t*, the probability of tuberculosis infection at age *a* resulting from either community or household transmission is


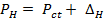


where *P_H_* is the cumulative probability of infection with *M. tuberculosis* from community or household transmission from birth to age *a*, *P_ct_* is the probability of infection from community transmission from birth to age *t*, and *∆_H_* is the probability of infection from age *t* to age *a* from either community or household transmission.

The difference in probability for a given age is


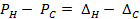


In this model, the probability of infection by age *a* is estimated as the cumulative proportion, or prevalence, of infection by age a, *P_H_* and *P_C_*.

Using a binomial model for infection [30], we define the age-specific prevalence for infection as the cumulative proportion of infection at age *a* and time *t* when the household is evaluated for tuberculosis or tuberculosis infection. We define the risk of infection with *M. tuberculosis* per year in the community as the annual risk of infection [31] and transform it to daily risk of infection, *r_c_*, for use in this model. For the household risk, we define the daily risk of infection in the household as the probability, r*_h_*, of acquiring infection with the specific strain of *M. tuberculosis* producing disease in the index case during a single day of living in the household. We also assume that transmission in the community and in the household represent independent, but not mutually exclusive, events and that individuals may be infected with more than one strain of *M. tuberculosis*.

The probability of acquiring at least one infection with *M. tuberculosis* from the community between age *t* and age *a* is


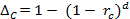


where *d* (*d* = *a – t*) is the duration of infectiousness in the index case. Similarly, the probability of becoming infected with the same strain as the index case at least once after *d* days of household contact, that is the SAR for tuberculosis infection, is


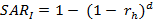


SAR of tuberculosis infection may be considered a fixed value within a given household assuming that members of that household have had a similar level of exposure to the index case through random mixing, that susceptibility to infection is similar among household contacts, and that virulence is similar among strains of *M. tuberculosis*.

Since infection acquired from the community is independent from infection acquired in the household, the probability of acquiring at least one infection from either source between age *t* and age *a* is


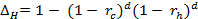


The difference in the cumulative proportion for tuberculosis infection is given by

|  | 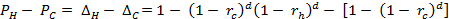  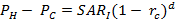 |  |
| --- | --- | --- |

The difference in prevalence is the product of the probability of escaping infection in the community from age *t* to age *a* and the probability of acquiring infection with a specific strain of *M. tuberculosis* through household exposure.

The prevalence difference in tuberculosis infection approaches the SAR_I_ for tuberculosis infection as either *r_c_* or the duration of infectiousness in the index case approaches zero. The prevalence difference will give a good estimate of SAR when *r_c_* is low, such as might be found in many industrialized countries with a low prevalence of tuberculosis or when transmission of a single strain is considered. Similarly, the prevalence difference will estimate the SAR_I_ when the period of infectiousness in the index case is short. Therefore, under these conditions


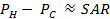


|  |  |  |
| --- | --- | --- |

We estimate the difference between the cumulative proportions using age-stratified samples taken from the population


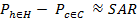


|  |  |  |
| --- | --- | --- |

where *h* is the subset of household contacts in the community at time *t* and *c* is the set of community members not living in households with index cases at time *t*. Age-specific and age-adjusted cumulative proportion differences were calculated for the contacts of index cases and community controls [29].


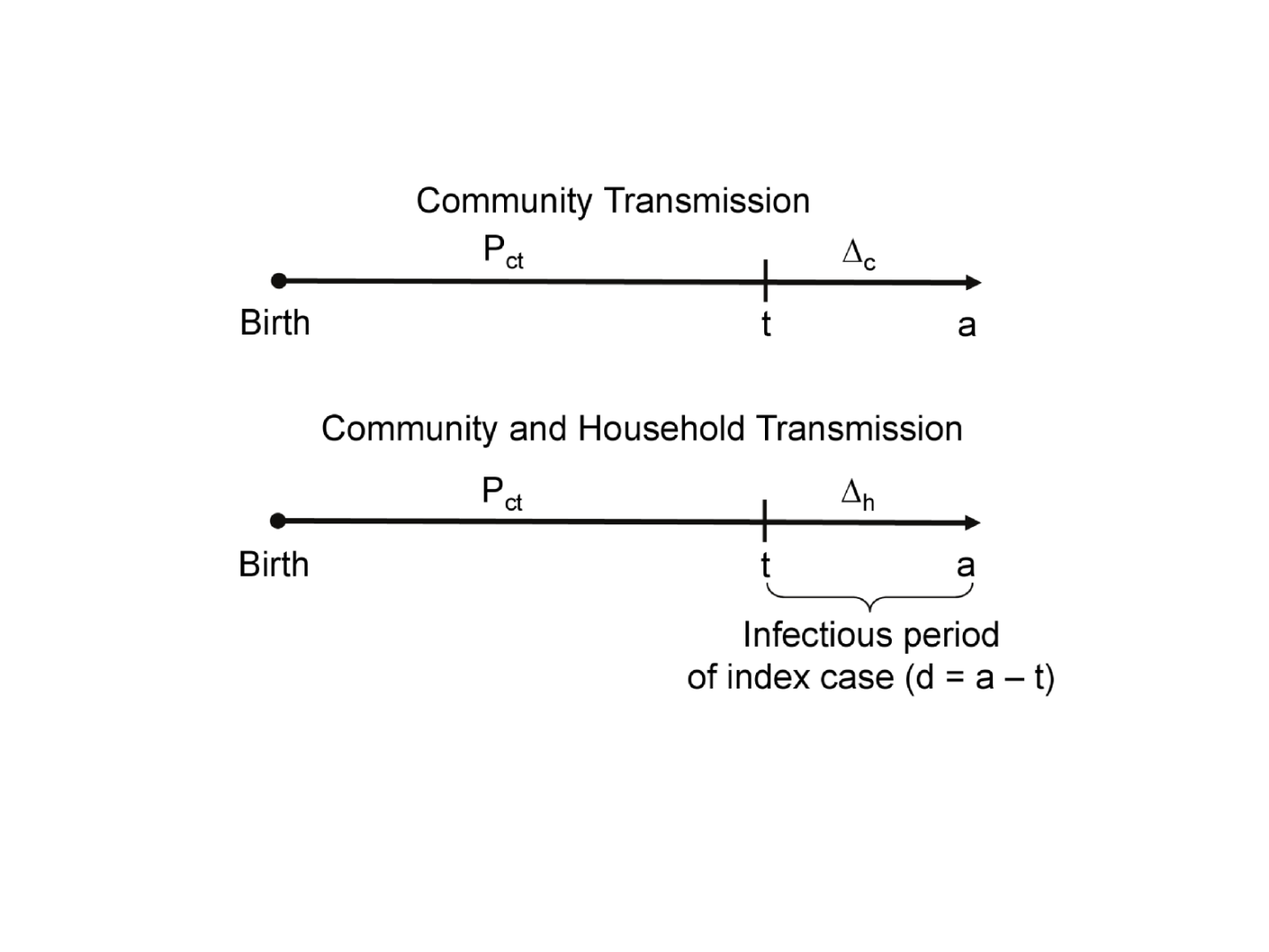


**Figure 1. Timeline for comparing the cumulative incidence of infection for persons living in a community and those who are also exposed to an infectious tuberculosis case.** The timeline represents the risk of tuberculosis infection from birth until age *a*, the age when an evaluation for tuberculosis infection is performed, for an individual with community transmission of infection and for an individual with either community or household transmission. In community and household transmission, the infection period of the index case begins at age *t*. The days of exposure (*d* = *a – t*) represent the duration of exposure to the infectious index case and the strain producing disease in the case.
